# Spleen Vagal Denervation Inhibits the Production of Antibodies to Circulating Antigens

**DOI:** 10.1371/journal.pone.0003152

**Published:** 2008-09-05

**Authors:** Ruud M. Buijs, Jan van der Vliet, Mari-Laure Garidou, Inge Huitinga, Carolina Escobar

**Affiliations:** 1 Instituto de Investigaciones Biomédicas, Universidad Nacional Autónoma de México, México, México; 2 Netherlands Institute for Neuroscience, Amsterdam, The Netherlands; 3 Departamento de Anatomía, Facultad de Medicina, Universidad Nacional Autónoma de México, México, México; University of Massachusetts Medical School, United States of America

## Abstract

**Background:**

Recently the vagal output of the central nervous system has been shown to suppress the innate immune defense to pathogens. Here we investigated by anatomical and physiological techniques the communication of the brain with the spleen and provided evidence that the brain has the capacity to stimulate the production of antigen specific antibodies by its parasympathetic autonomic output.

**Methodology/Principal Findings:**

This conclusion was reached by successively demonstrating that: 1. The spleen receives not only sympathetic input but also parasympathetic input. 2. Intravenous trinitrophenyl-ovalbumin (TNP-OVA) does not activate the brain and does not induce an immune response. 3. Intravenous TNP-OVA with an inducer of inflammation; lipopolysaccharide (LPS), activates the brain and induces TNP-specific IgM. 4. LPS activated neurons are in the same areas of the brain as those that provide parasympathetic autonomic information to the spleen, suggesting a feed back circuit between brain and immune system. Consequently we investigated the interaction of the brain with the spleen and observed that specific parasympathetic denervation but not sympathetic denervation of the spleen eliminates the LPS-induced antibody response to TNP-OVA.

**Conclusions/Significance:**

These findings not only show that the brain can stimulate antibody production by its autonomic output, it also suggests that the power of LPS as adjuvant to stimulate antibody production may also depend on its capacity to activate the brain. The role of the autonomic nervous system in the stimulation of the adaptive immune response may explain why mood and sleep have an influence on antibody production.

## Introduction

Infection is an acute danger for body homeostasis that needs to be neutralized by a well coordinated action of multiple organs of the body. In addition to a primary role for the immune system, recently a vital function for the central nervous system in the defense mechanisms of the body has been shown[1], [2]. The first line of defense to infection is formed by cells of the innate immune system that detect e.g. microbes and initiate a local inflammatory response[3]. This inflammation is signaled to the central nervous system (CNS) by humoral and sensory routes[4], [5]. The reaction of the brain is multifaceted and immediate; it induces the secretion of anti-inflammatory corticosteroids, changes the body temperature and releases vagal neurotransmitters that serve to suppress the inflammation by diminishing the release of tumor-necrosis factor TNF[3], [6]. The second defense line against invading microbes is the initiation of antigen-specific T- and/or B-cell mediated adaptive immune response that is specifically directed against the invader [7], [8]. The involvement of the brain in this adaptive immune response is not well understood. Therefore we investigated a possible bidirectional interaction of the brain with the spleen via the autonomic nervous system using four different approaches. 1. We demonstrated the capacity of the brain to communicate with the spleen using pseudo rabies virus (PRV) retrograde tracing and showed that the spleen receives not only sympathetic input but also parasympathetic input of which the latter motor neurons are located in the brain stem. 2. Since the spleen is mainly monitoring the blood for circulating antigens[9] we investigated whether by intravenous (i.v.) trinitrophenyl-ovalbumin (TNP-OVA) injection the induction of specific antibodies occurred. I.v. injection of TNP-OVA alone was not sufficient to induce specific antibodies to TNP-OVA nor did it induce an activation of the brain as measured by the absence of induction of c-Fos and corticosterone release. 3. However signals of a clear activation of the brain were observed when we combined i.v. TNP-OVA with LPS. A pronounced increase in corticosterone secretion was associated with the induction of c-Fos in the same areas of the brain that provide parasympathetic autonomic information to the spleen. Following these demonstrations of brain activation three days later TNP-specific IgM were detected in the circulation. 4. We hypothesized that the presence of LPS activated neurons in these locations, suggested a feed back circuit between brain and immune system, whereby the cytokines released after infection activate autonomic centers in the brain resulting in enhanced parasympathetic output. Consequently we investigated the interaction of the brain with the spleen and studied the induction of antibodies following specific denervation of the spleen either sympathetically of parasympathetically. Subsequently following LPS + TNP-OVA injection we observed that specific parasympathetic denervation but not sympathetic denervation of the spleen eliminated the LPS-induced antibody response to TNP-OVA.

These results demonstrate that the immune system and the brain interact whereby the activation of the brain by LPS/cytokines and the subsequent activation of the parasympathetic output of the central nervous system are able to induce an immune response. These observations agree very well with those obtained by the group of Tracey[2] whereby the parasympathetic output of the brain is essential to contain the inflammation. The present data indicate that at the same time the parasympathetic output induces the immune system to react with the production of specific antibodies against the invading substance. This extended role of the central nervous system may provide for the first time an explanation why mental condition might be important for the efficiency of the body to fight an infection[10], [11].

## Results

### Autonomic innervation of the immune system

**Figure 1:**
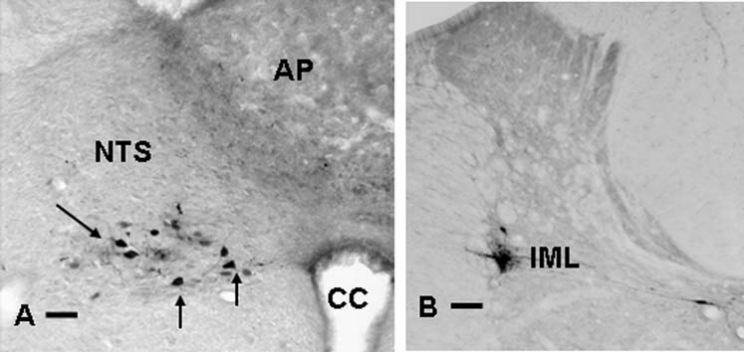
PRV labeling from spleen shows parasympathetic and sympathetic autonomic neurons in brain stem (A) and spinal cord (B). After injection of PRV into the spleen, the origin of the parasympathetic respectively sympathetic input to the spleen is revealed. A) PRV positive neurons are present in the neurons of the dorsal motor nucleus of the vagus (DMV), at this stage of infection there are no labeled neurons in the area postrema(AP) or nucleus tractus solitarius (NTS).B) PRV positive neurons are present in the sympathetic intermediolateral column (IML) or in the area of the central canal. (Bar = 50 µm).

We initially examined whether the brain is connected with the immune system via the two different branches of the autonomic nervous system (ANS), or whether it employs only sympathetic innervation for its communication with the spleen. The trans-synaptic retrograde tracer Pseudorabies virus (PRV)[12] was injected into the spleen. After two day survival the first PRV positive neurons were visible in sympathetic motor neurons in the intermedio lateral (IML) column of the spinal cord, in addition at the same time also some PRV positive neurons were visible in the dorsal motor nucleus of the vagus (DMV) (Figure 1), illustrating that indeed the brain has the anatomical connections to affect the spleen by the two branches of the ANS.

**Figure 2:**
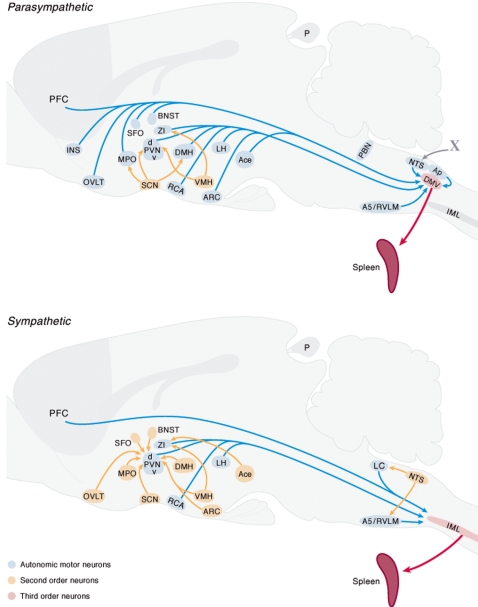
Distribution of pseudo rabies virus (PRV) labeled neurons in different areas of the brain following injection of PRV into the spleen after sympathetic A) or parasympathetic denervation B). Following different survival times these procedures revealed respectively the first (red) or second order (blue) or third order (brown) neurons that project to the spleen. The upper graph illustrates the areas in the brain that have parasympathetic (pre)autonomic neurons and the lower illustrates those areas that have sympathetic (pre)autonomic neurons.

In successive experiments using the same technique but longer survival times we investigated which areas of the brain can communicate with the spleen and examined the distribution of neurons in the central nervous system that are connected with the spleen either via the parasympathetic or the sympathetic autonomic nervous system. Hereto we denervated the spleen locally, respectively sympathetically (SSX) or parasympathetically (PSX) and used the PRV tracing technique to test our surgical approach in order to denervate the spleen selectively and consistently. Two days after injection of PRV in the SSX spleen, labeling of neurons in the DMV only was observed, with no detection of neurons in the IML. Two days after PSX, PRV labeled neurons were only present in the IML and not in the DMV. Longer survival times after PSX or SSX resulted in the visualization of different areas in the brain stem and higher brain regions that contain pre-autonomic neurons that project to the DMV in agreement with previous findings analyzing the autonomic connections of the brain with the pancreas or liver[13], [14] a lesser number of brain regions project directly to the sympathetic motor neurons.(Figure 2.) In the hypothalamus only the paraventricular nucleus, the zona incerta and the lateral hypothalamus contain neurons that project directly to parasympathetic or sympathetic motor neurons. Interestingly all circumventricular organs contain neurons that project directly only to parasympathetic motor neurons, furthermore also the central nucleus of the amygdala and the medial preoptic nucleus project directly to the DMV.

In the next phase of our experiments we used this selective denervation to examine whether the brain may use these autonomic connections to modify the immune response of the spleen. Since previous studies demonstrated a role of the parasympathetic innervation in the innate immune response[1], [2], [15] we hypothesized that the brain may also affect the adaptive immune response by its autonomic output.

### Brain activation is associated with the adaptive immune response

**Figure 3:**
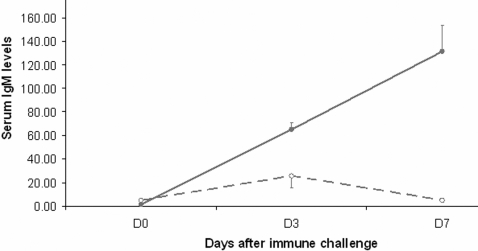
Specific IgM antibodies to TNP-OVA are only detected in the circulation after an i.v. injection of TNP-OVA with a prior injection of LPS. The antibody response was only present when LPS preceded the injection of TNP-OVA(uninterrupted line). Dashed line shows the IgM levels in plasma after injection of TNP-OVA only. (Two way ANOVA indicated difference between TNP-OVA saline and TNP-OVA + LPS p<0.01, Tukey post hoc test only showed significant difference between D0 and D3 and D7 after TNP-OVA+LPS injection p<0.001).

**Figure 4:**
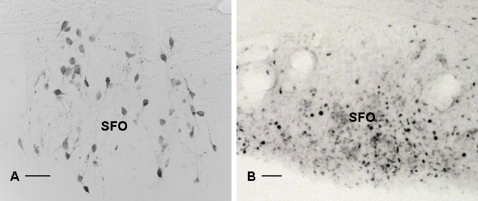
The same area in the brain contains pre-autonomic neurons projecting to the spleen and neurons that are activated after LPS injection. The presence of pre-autonomic spleen projecting neurons in the subfornical organ (A) is detected after PRV injection in the spleen. After i.v.injection of LPS, activated neurons are detected by c-Fos immunocytochemistry in the SFO (B) Bar  = 50 µm.

**Figure 5:**
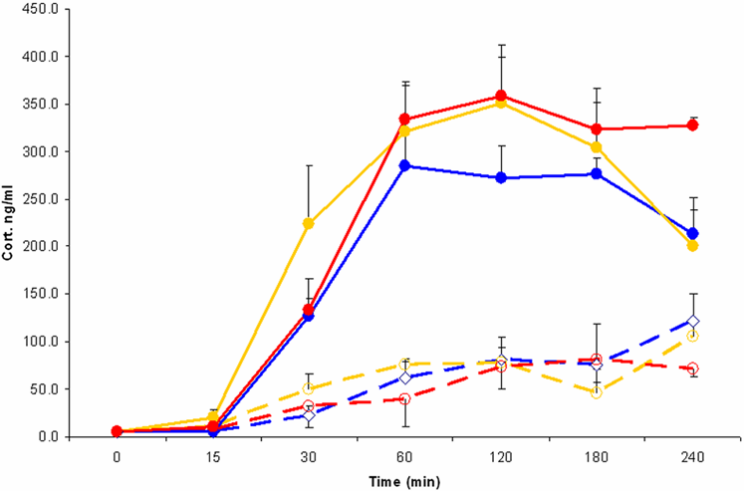
Shows the response of corticosterone after an injection of TNP-OVA alone (dashed lines) or TNP-OVA+LPS in sham (blue) or SSX (yellow) or PSX (red) animals. Clearly LPS induces the secretion of corticosterone but no difference can be observed between denervated or intact animals, illustrating that after i.v. injection of LPS the corticosterone response is not dependent on sensory autonomic information from the spleen. (Two way ANOVA indicated difference between the LPS treated groups with all other groups without LPS p<0.01. Tukey post hoc test showed significant difference of day 7 and 10 of the sham and sympathetic denervation as compared to all other time points p<0.001.)

Since the spleen is mainly involved in monitoring the general circulation for foreign substances[9] we investigated the immune response by injecting intravenously (i.v.) tri-nitro-phenol coupled to ovine albumin (TNP-OVA). At the same time we investigated whether this protocol would activate the brain. Hereto animals received more than a week previous to the injections an intra jugular catheter for stress free intravenous injection and blood withdrawal[16]. Six animals were used to examine the activation of the brain and at six time points after the injection blood was taken to measure the response of corticosterone. The animals were subsequently sacrificed 240 minutes after the injection of TNP-OVA to analyze which areas of the brain were activated by the circulating TNP-OVA using staining for the immediate early gene c-Fos. The other six animals were used to measure 3 and 7 days after the injection, circulating antibodies to TNP-OVA. The results showed that injection of TNP-OVA alone did not result in any detectable increase in blood corticosterone or in c-Fos staining in the brain as compared to saline injected rats, indicating the absence of neuronal activation. Animals of the same group showed 3 and 7 days later no IgM against TNP-OVA demonstrating an absence of the specific immune response as well; indicating that i.v. injected TNP-OVA is not immunogenic *per se* (Fig. 3). In view of the fact that lipopolysaccharide (LPS) is used as an adjuvant in order to induce the production of antibodies[17], we aimed to induce an immune response to i.v. injected TNP-OVA by using a prior injection of LPS. Hereto a minimal dose of LPS was i.v. injected 30 minutes prior to the injection of TNP-OVA and the animals were subjected to the same protocol as for TNP-OVA only. The 30 min time point was chosen since corticosterone measurements indicated an initial activation of the hypothalamo-pituitary-adrenal system 30 minutes after the injection of LPS. In addition also the behavioral response of the animals started 30 minutes after the injection. After LPS + TNP-OVA injection, animals perfused 3 hours after the injections did show c-Fos in many areas of the brain as evidence of brain activation in addition also a strong corticosterone response could be observed already 30 minutes after the injection of LPS (Fig. 4 and 5). Finally also after 3 and 7 days a significant level of anti-TNP IgM was detected (Figure 3).

### LPS induces activation of brain sites that connect to the spleen

The detection of antibodies to TNP-OVA confirmed that LPS as an adjuvant indeed facilitates an adaptive immune response, that, as demonstrated by the detection of c-Fos in the brain, coincided with the activation of the CNS. The activation of the brain was further confirmed by the fact that also blood corticosterone levels were increased after LPS and TNP-OVA injection. Remarkably, all the circumventricular organs (CVO's) of the brain; the area postrema, the organum vasculosum of the lamina terminalis and the subfornical organ, demonstrated signs of neuronal activation by revealing neurons containing c-Fos after i.v. LPS injection. CVO's are areas of the brain where the blood brain barrier is less effective and where information of the blood may be signaled to specialized neurons[18]. This observation confirmed earlier studies that showed LPS injection to coincide with strong but very selective activation of regions in the brain where specialized cell bodies can sense directly the composition of the blood [5], [19], [20]. At this moment it is not clear whether LPS directly or the LPS induced cytokines are responsible for this neuronal activation[21]. More importantly the present PRV tracing experiments showed that these CVO's and most other brain areas that are activated after LPS, project via the autonomic motor neurons to the spleen. (See figure 2, 4). These observations suggest that LPS induces directly or indirectly an activation of brain sites that have autonomic connections with the spleen which may affect the function of the spleen.

In order to examine whether the LPS induced activation of the brain is dependent of the possible interaction of LPS with vagal nerve terminals in the spleen and thus could be the result of sensory stimulation of autonomic nerve terminals in the spleen, we examined the c-Fos response in intact and SSX and PSX denervated animals. In none of these groups any difference in c-Fos activation of the brain was noted as compared to intact animals. In the NTS, an area where autonomic visceral feedback is concentrated[22], the number of c-Fos stained neurons remained constant around 100 c-Fos neurons per section in the NTS in the three groups of animals (see supplemental data for more details). In addition the LPS induced corticosterone response of spleen denervated animals did not differ from the intact group (Figure 5) indicating that the sensing of LPS (or the sensing of induced cytokines) by the brain had not been altered by the denervation of the spleen. We take these two observations as evidence that the brain is affected by LPS or cytokines in the general circulation and not necessarily needs the connection with the spleen to become activated. This concurs with other experimental evidence where LPS could still activate the brain also after complete vagal denervation.[23], [24].

**Figure 6:**
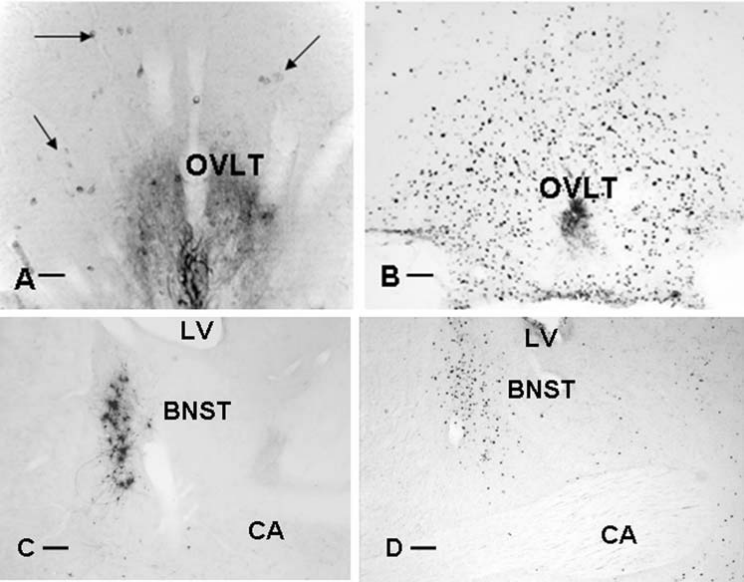
Illustrates neurons in the organum vasculosum lamina terminalis (OVLT) (A-B) and bed nucleus of the stria terminalis (BNST) (C-D) containing c-Fos 3 hours after injection of LPS in the general circulation (B,D) or PRV 4 days after injection in sympathetic denervated animals (A,C). These comparable sections of the same areas show that these areas not only are activated after LPS injection, but that they also have the capacity to communicate to the spleen via the vagus Bar  = 50 µm.

Consequently the present data suggest that circulating LPS or cytokines may indeed affect the brain mainly via the CVO's [4], [5], [20]. Since the present experiment showed that LPS induces c-Fos in many areas in the brain such as the paraventricular nucleus, amygdala, bed nucleus of the stria terminalis (BNST)etc. that receive direct input from the circumventricular organs[18], [25], [26] it seems plausible that from these “windows of the brain” the rest of the CNS is activated. The present PRV retrograde tracing experiments after SSX showed that especially the parasympathetic autonomic motor neurons in the DMV get direct input from neurons in the CVO's and from other structures such as the NTS and BNST that show c-Fos activation after LPS injection (Figure 6). This observation confirms earlier anatomical studies that showed that the AP receives direct input from the DMV[26], and extends it to the sub fornical organ and organum vasculosum of the lamina terminalis (Figure 4). Their connection with the DMV and their activation by circulating LPS brings e.g. these CVO's in a unique position to affect the autonomic parasympathetic output to the spleen.

### Connection of the brain with the spleen is essential for the adaptive immune response

**Figure 7:**
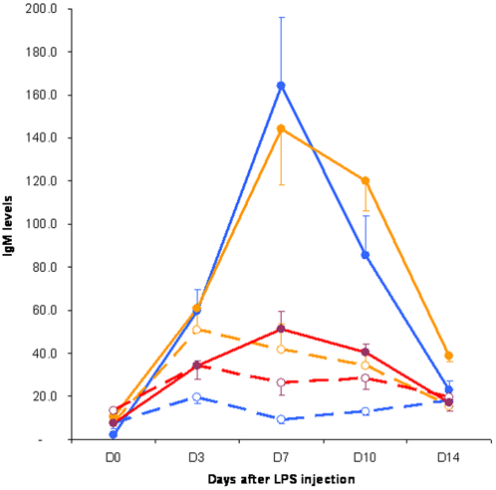
Illustrates absence of TNP-OVA IgM antibody production after parasympathetic denervation of the spleen. TNP-OVA IgM antibody production after i.v. injection of saline and TNP-OVA (dashed lines) or LPS and TNP-OVA(uninterrupted lines) in animals that sustained a parasympathetic denervation (red) or a sympathetic denervation (yellow) of the spleen or were sham operated (blue). Only parasympathetic denervation results in a nearly complete loss of antibody production with a similar response as if no LPS were given. (Two way ANOVA indicated difference between the sham and sympathetic denervation + LPS with the other groups without LPS and the parasympathetic denervated group with LPS p<0.01. Tukey post hoc test showed significant difference of day 7 and 10 of the sham and sympathetic denervation as compared to all other time points p<0.001.)

Consequently we investigated whether the autonomic output of the brain might be involved in the adaptive immune response and thus to what extend information from the brain to the spleen is essential for the generation of antigen specific antibodies. Hereto we investigated the induction of antibodies to TNP-OVA using LPS as adjuvant using the same i.v. procedure as before but disconnected the spleen from the brain by PSX or SSX at least one week prior to the i.v. injection of TNP-OVA with 10 µg/100 g LPS in intact and in denervated animals. Blood was collected 3, 7,10 and 14 days after immunization and the antibody response examined by measuring TNP-OVA specific IgM with ELISA. As observed before (figure 3), intact animals showed a clear increase in TNP-OVA specific IgM antibodies 3, 7 and 10 days after the injection, while animals where the parasympathetic innervation of the spleen was removed had a severely diminished (-70%) immune response (Figure 7). In contrast sympathetic denervation did not affect the production of TNP-OVA-specific antibodies, which can be seen as additional evidence of the specificity of the stimulatory effect of the parasympathetic system on antibody production. The observation that PSX or SSX did not change or diminish the c-Fos induction in the brain or the corticosterone levels upon injection of LPS illustrates that the diminishment of antibody production after PSX only is not due to a change in the sensing of the LPS.

## Discussion

The present observations have several implications for our understanding of how the central nervous system may be involved in the organization of the immune response. Recent studies of the group of Tracey[1], [2], [6] have shown that acute inflammation is actively suppressed by vagus nerve output and that consequently the CNS diminishes the harmful effects of inflammation. At first sight it may seem contradictory that the vagus holds down inflammation while here we show that activation of the dorsal motor nucleus of the vagus by inflammatory substances is essential for the induction of a specific antibody response. However we have to consider that the present study together with the data of Tracey shows that *after* infection and subsequent inflammation the aim of the brain is twofold protective: to contain both the inflammation as a consequence of the infection and subsequently to induce antigen specific memory within the immune system to prevent new infections. Consequently the picture emerges of a multitude of actions that are taken by the brain after the detection of infection. These actions can be divided in two types of measures one via the hormonal system and one via the autonomic nervous system. The secretion of hormones (e.g. corticosterone) may serve to counteract the inflammatory and metabolic disturbances induced by pro-inflammatory cytokines. The brain can direct the autonomic response to the site of the infection, resulting in local vasodilatation while in the rest of the body, skin blood vessels are constricted to raise core body temperature; it also includes an acute increase on muscle glucose uptake which is also neurally mediated[27] and the organization of the suppression of the innate immune response locally, by well directed output of the fibers of the parasympathetic system[2]. At the same time, the present study indicates that the activation of the parasympathetic system by inflammation boosts the production of specific antibodies; logically this will take place locally at the sites of the immune organs. At present the mechanism of interaction of the vagus with the spleen in order to facilitate the immune response is not known. A recent study of the group of Tracey[28] suggests by the absence of choline acetyltransferase or vesicular acetylcholine transporter staining in nerve terminals that the vagus nerve interacts with the Celiac-superior mesenteric plexus ganglion in order to convey the vagus message to the spleen. However the absence of the classical vagus transmitter acetylcholine is not sufficient proof for the absence of direct input from the vagus. For example several studies have shown the presence of vagal innervation of the liver[29], [30] while also in the liver choline acetyltransferase or vesicular acetylcholine transporter staining is absent[31]. Although by lesioning different nerve branches entering the spleen we could identify which nerves carry the vagal input to the spleen we were not able to identify what structures the vagal nerve terminals target in the spleen. The fact that we were able to demonstrate labeled neurons in the DMV after sympathetic denervation illustrates that DMV labeling does not arise from labeling via the celiac-superior mesenteric plexus ganglion. Further studies using different tracing techniques will have to demonstrate where actually the vagal nerve terminals end in the spleen and what transmitter is used. Possibly the vagus influences the uptake, processing and presentation of antigens by dendritic cells to B-cells, or T helper cells to B-cells. At present we do not have the evidence that at the same time all lymphoid organs will be activated upon intravenous infection or that the parasympathetic output to the lymphoid organs is regionally organized just as the output to other organs of the body[32]. However it seems likely that the obvious and main advantage to give the brain such an organizational role in the immune response is that also by localized infections the alerting message will be spread to other immune organs than those close to the site of the infection. Another important aspect of the present study is that it illustrates that the adjuvant effect of LPS on antibody formation is not only an effect of LPS or LPS-induced cytokines on the immune system, but largely an effect on the brain which induces the augmentation of the formation of antibodies by the immune system. This coherent role for cytokines to act on the brain after which the brain alerts the immune system, may offer an explanation for the fact that one of the co-morbidities of obesity is higher levels of antibody production and a higher frequency of autoimmune disease whereby leptin as well as other cytokines released by fat tissue may have the role of stimulating the brain. [33]–[35]

Moreover the important role for the brain in fighting the consequences of infection may provide an explanation why mood, sleep and stress may affect the immune response of the body[36].

## Materials and Methods

Retrograde tracing from the spleen was achieved by injecting 2 µl of pseudo rabies virus (PRV) of the Bartha strain (containing 10^8^ plaque forming units) into the spleen. See for details[12], [13] . Controls consisted of PRV injections in the spleen of animals that prior received complete sympathetic and parasympathetic denervation of the spleen, these controls revealed no sign of infection in the brain or spinal cord demonstrating the specificity of the procedure. Detection of PRV or c-Fos was executed by immunohistochemistry according to published methods[13].

Analysis of c-Fos, corticosterone was achieved by injecting free moving non anaesthetized animals intravenously via a jugular catheter[16] with 10 µg/100 g LPS with or without 15 µg TNP-OVA/100 g in 0.5 ml saline. For c-Fos the animals were sacrificed 3 hours after the injection of TNP-OVA with or without LPS. For corticosterone measurement the blood was collected just before the injection and after 15, 30, 60, 120, 180 and 240 minutes. Based on the behavioral response of the animals and the increase of corticosterone after 30 minutes it was decided to induce antibodies specific for TNP-OVA by injecting animals first with 10 µg/100 g LPS (or with saline as control) followed after 30 minutes by 15 µg TNP-OVA/100 g. For antibody measurement blood was collected before the injection and 3-7-10 and 14 days after the injection of TNP-OVA.

Plasma corticosterone was measured by a radioimmunoassay kit of ICN Biomedicals (Costa Mesa CA). Specific IgM for TNP-OVA were measured by initially isolating the specific TNP-OVA IgM from plasma using Elisa plates coated with TNP-OVA, whereupon the bound IgM were measured using the rat IgM detection kit of Bethyl laboratories (cat nr.E110-100) as standard a pooled plasma was used of rats that were immunized with TNP-OVA.

### Chirurgical sympathetic denervation of the spleen (SSX)

**Figure 8:**
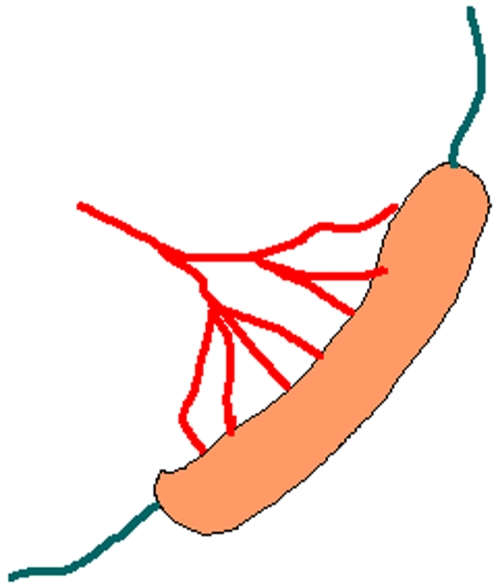
Schematic representation of the innervation of the spleen. The sympathetic input (red) reaches the spleen via the arteries, the parasympathetic input (blue) reaches the spleen via both tips of the spleen.

For all operations rats were anaesthetized using a mixture of Hypnorm (0.05 ml/100 g body weight, i.m.) and Dormicum (0.04 ml/100 g body weight, s.c.). During abdominal surgery, the abdominal cavity was bathed regularly with saline to prevent drying of the viscera. The wound was closed with nontraumatic sutures. All animal experiments were conducted according to the guidelines of the European community and with the approval of the local animal care committee. After a midline incision the stomach was pushed up and to the right. Thus the blood vessels to and from the spleen are revealed. After the arterial branch to the stomach a bifurcation indicates the first branching point of the arterial supply to the spleen (Figure 8), here the artery usually is covered by lymph nodes. From this bifurcation on the arteries will split many times and end at the hili of the spleen. Sympathetic nerves run along and around these arteries to reach the spleen, the area just before and after the bifurcation was chosen to remove the sympathetic nerves; the nerve bundles were removed using micro-surgical instruments under an operating microscope (25× magnification). To ensure that at the end of the operation all the small pieces of nerve tissue around the artery are removed completely, a solution of 37% formaldehyde was applied for 1 minute on the surface of the artery. Finally this procedure results in a complete sympathetic denervation of the spleen as proven by retrograde PRV tracing and the absence of staining in the DMV.

### Selective parasympathetic denervation of the spleen (PSX)

The parasympathetic nerves enter the spleen at both tips, (Figure 8) therefore during the surgery these tips are sequentially exposed in order to be able to cut the nerves. Hereto a midline incision is made and the spleen pulled a little towards the site of the incision.

Then the nerve at the tip of the spleen is cut. If the nerve is not visible because connective tissue blocks the view, the connective tissue should be removed and herewith the nerve. Also remove the connective tissue between the tip and the first hilus because some parasympathetic input reaches the spleen via this connective tissue. In order to reach the deeper lateral part of the spleen, the spleen is further pulled towards the midline to reach this lower tip of the spleen. The nerve is visualized and followed back to the plexus, then the connective tissue removed (with the nerves) from this plexus back to the spleen. In addition to finalize the removal of all parasympathetic nerves, the connective tissue between the hilli is removed except around the vessels that are encircled by the sympathetic nerves. This procedure results in a complete parasympathetic denervation of the spleen, as proven by retrograde PRV tracing, the presence of neurons in the dorsal motor nucleus of the vagus (DMV) and the absence of staining in the sympathetic motor neurons in the IML.
